# Supplementary figures and images for: Species-, organ- and cell-type-dependent expression of SPARCL1 in human and mouse tissues
Source: PLoS One. 2020 May 21;15(5):e0233422. doi: 10.1371/journal.pone.0233422 (PMC7241726; doi:10.1371/journal.pone.0233422)

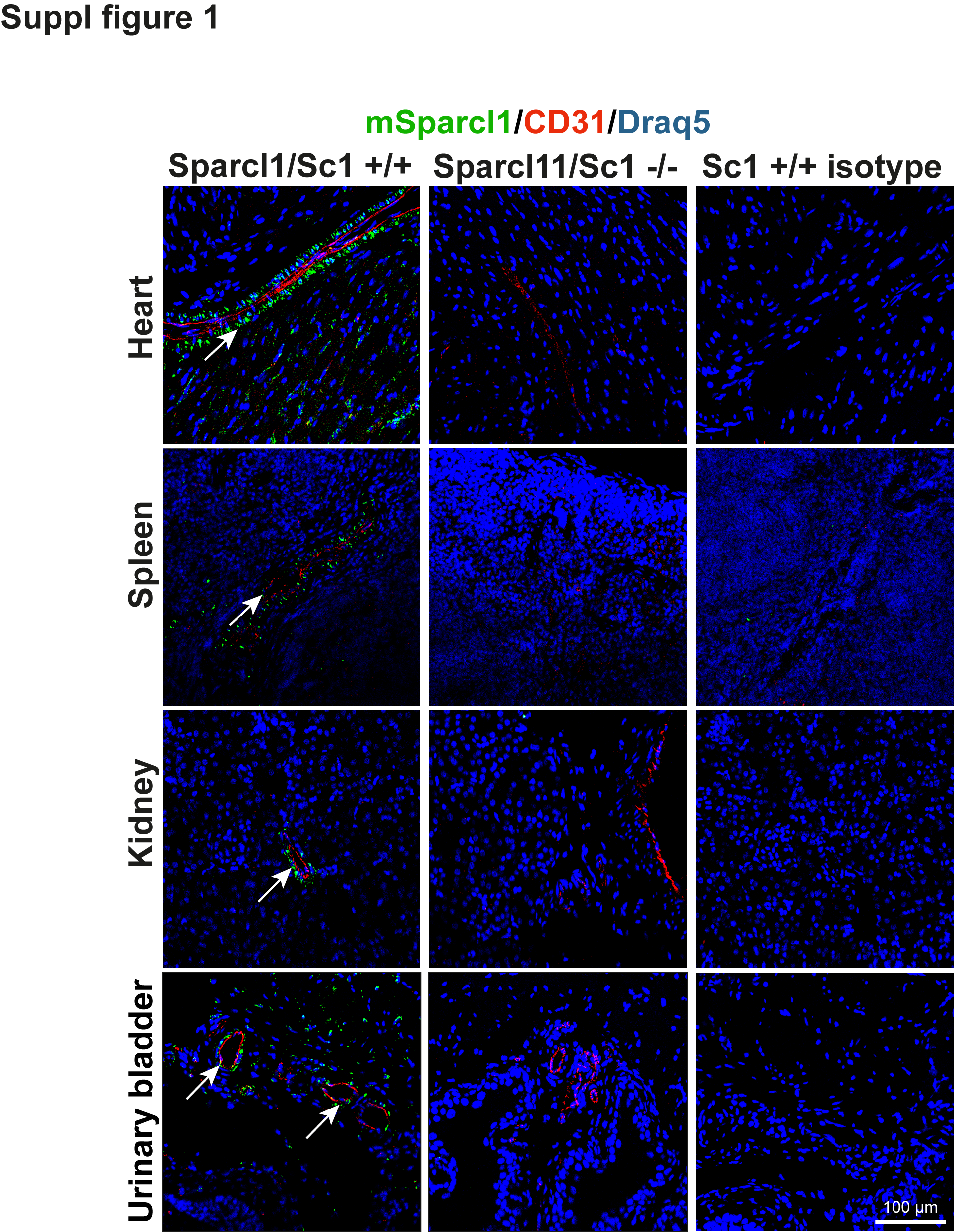

Supplement: S1 Fig — Murine Sparcl1 expression (Sc1 +/+, green, arrows) was determined by immunofluorescence in various organs of Sparcl1 wild-type mice (n = 3). Isotype antibody (Sc1 +/+ isotype) and knockout mouse (Sc1-/-, n = 3) staining were used as controls. All tissues were counterstained using DRAQ5 (blue). Scale bar = 100 μm. (TIF) [file pone.0233422.s001.tif]

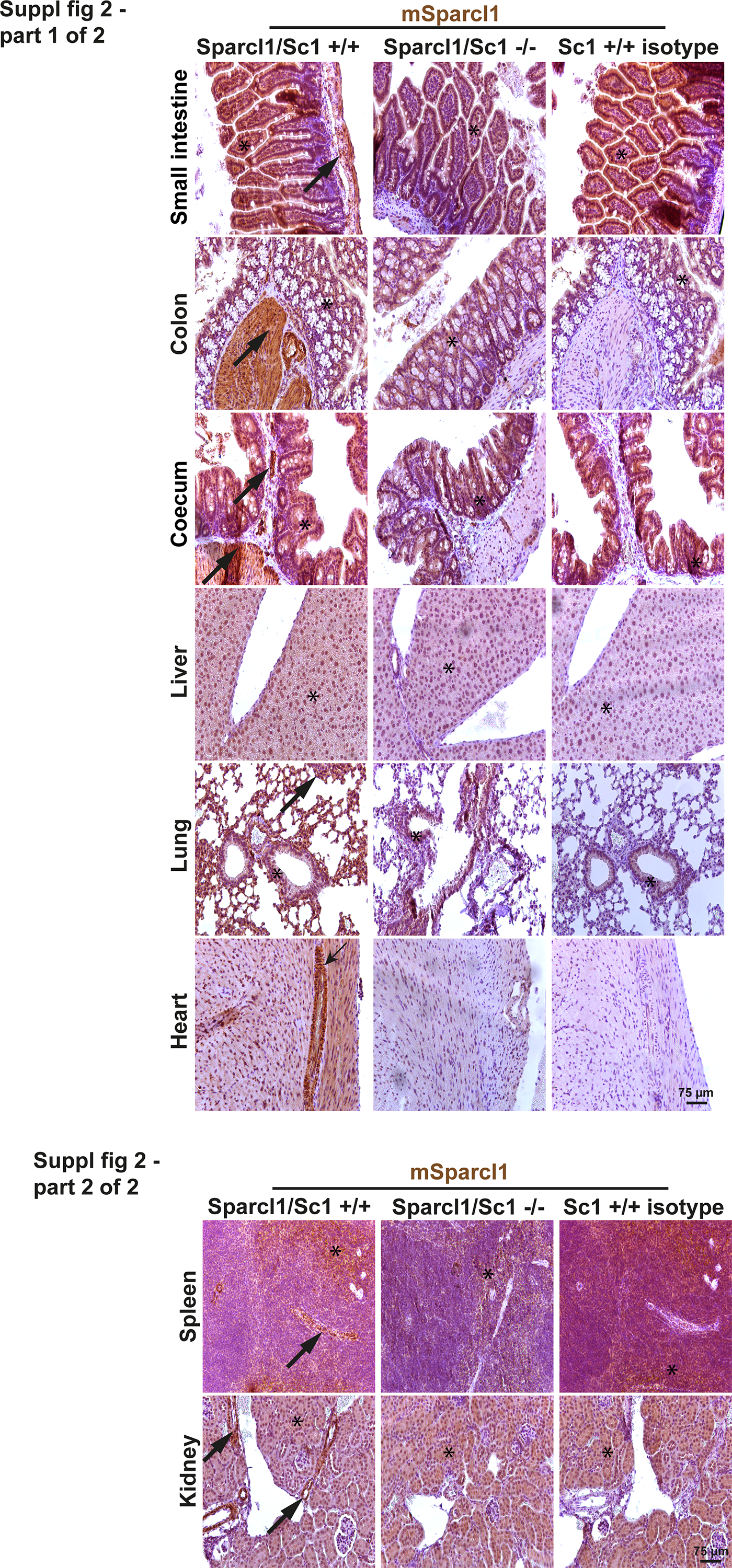

Supplement: S2 Fig — Murine Sparcl1 expression (Sc1 +/+, brown, arrows) was determined by permanent IHC in various organs of Sparcl1 wild-type mice (n = 3). Isotype antibody- (Sc1 +/+ isotype) and knockout mice (Sc1 -/-, n = 3) staining were used as controls. Asterisks indicate nonspecific staining. Scale bar = 75 μm. (TIF) [file pone.0233422.s002.tif]

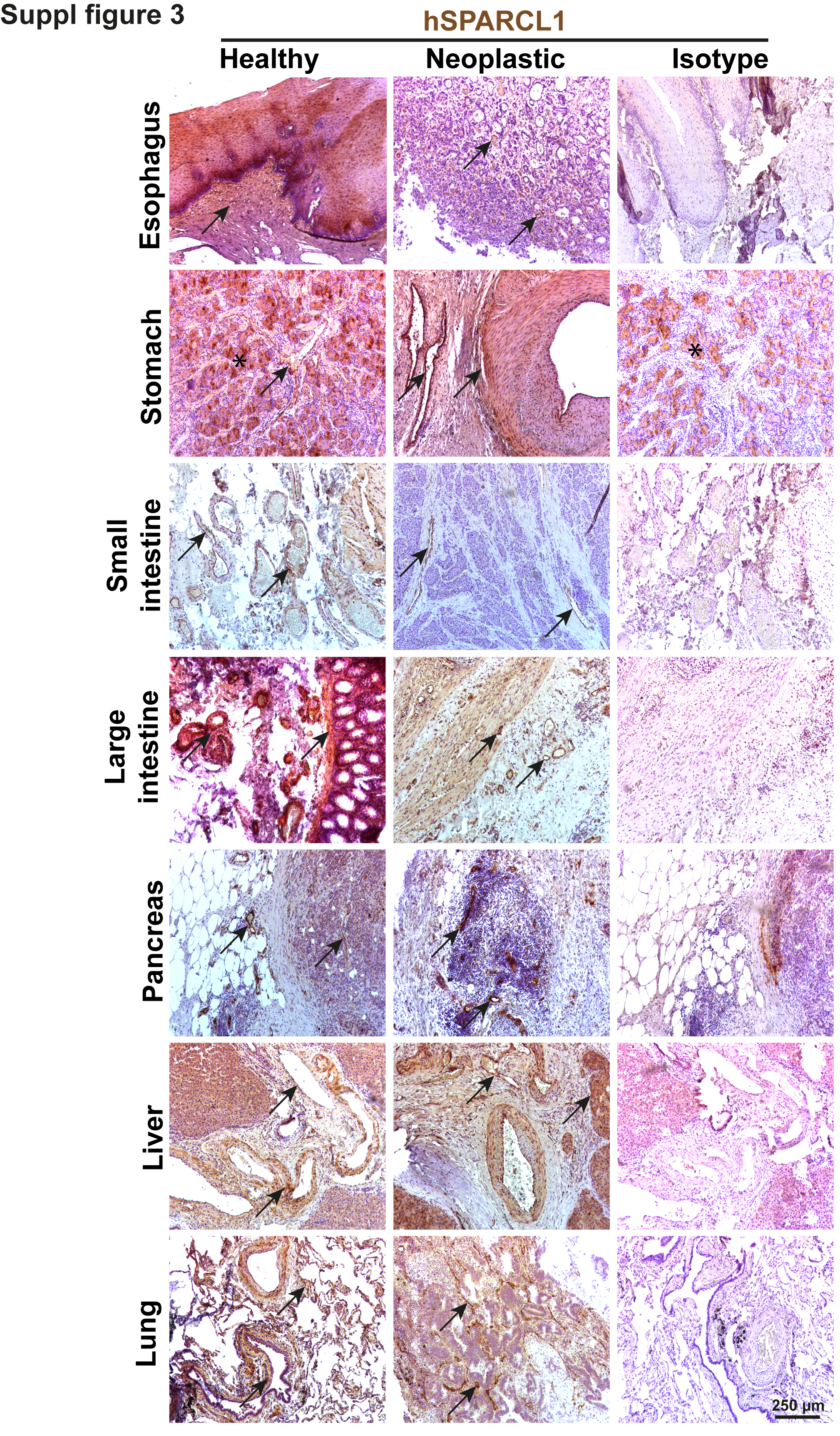

Supplement: S3 Fig — Human SPARCL1 expression (brown, arrows) was determined by permanent IHC in various healthy and neoplastic organs of human patients (healthy, neoplastic, n = 3 for all organs except oesophagus, small intestine and lung tumor with n = 2). Isotype antibody staining of consecutive sections was used as a negative control (isotype). Asterisks indicate nonspecific staining. Scale bar = 100 μm. (TIF) [file pone.0233422.s003.tif]
