# Supplementary material for: Species-, organ- and cell-type-dependent expression of SPARCL1 in human and mouse tissues
Source: PLoS One. 2020 May 21;15(5):e0233422. doi: 10.1371/journal.pone.0233422 (PMC7241726; doi:10.1371/journal.pone.0233422)
Supplement: Attachment — Submitted filename: plosone-Review.pdf [file pone.0233422.s005.pdf]

Klingler Anika et al show that SPARCL1 expresses differently among species, organ and cell types. The experiments are well designed and performed with different controls. Relying on this study, we should be careful when we want to transfer findings from mouse models for the human patients. And some drug screen dependent on mice may not be applied into human diseases. It should be accepted for this journal and I have some concerns:

The major one is:

For human SPARCL1, a western blot is needed for its expression in each tissue and the migrating bands should be included in the WB. I am interested that the migrating bands may be functional.

And some minor concerns:

1) In Fig1B, migrating bands are different between Colon and Coecum, the authors should give an explanation for this.

2) Similar as the above one, the authors show that human SPARCL1 is downregulated in malignant diseases, a WB is needed to test whether there is difference in the migrating bands.

3) SPARCL1 in mouse should be written as Sparcl1, with only the first letter capitalized.
