# Supplementary material for: Species-, organ- and cell-type-dependent expression of SPARCL1 in human and mouse tissues
Source: PLoS One. 2020 May 21;15(5):e0233422. doi: 10.1371/journal.pone.0233422 (PMC7241726; doi:10.1371/journal.pone.0233422)
Supplement: Attachment — Submitted filename: comments on PONE-D-20-06477.doc [file pone.0233422.s004.doc]

SPARCL1 is a matricellular protein with tumorigenic function and is frequently down-regulated in tumors. Previous studies have found that SPARCL1 is differentially expressed in liver between mice and human, which is probably associated with colorectal carcinoma and its metastasis to liver. However, whether and how SPARCL1 is expressed in all organs and in other species has not been investigated. In the present study, the authors systematically analyzed the expression of SPARCL1 in multiple organs with various methods and compared the expression of SPARCL1 between mouse and human. And the results shows that in human samples, SPARCL1 was detected in all organs with the strongest expression in stomach, large intestine and lung, which is consistent with the expression pattern in mice; but murine liver showed an absence of SPARCL1 expression that is different with human liver, indicating that SPARCL1 function may not be similar in all tissues but dependent on species. The experiments were well designed, and were carefully controlled by setting SPARCL1 complete knock-out mouse and using healthy tissue and respective tumor tissue of the same organs. The results were clear and cautiously interpreted and the conclusion seems to be convictive. Generally speaking, this is an interesting manuscript regarding the expression of SPARCL1. However, revisions are needed before it could be accepted for publication in *PLOS ONE*.

Major points:

1. The main purpose of the study was to widely compare the expression of between murine model and human tissue. Therefore the author should focus on this purpose. It is acceptable to compare the SPARCL1 expression between different organs, different cells and between normal and neoplastic tissues, and the results are significant and interesting. However, during writing of this manuscript, it should always focus on the purpose of comparing the expression of between murine model and human tissue. Therefore the writing needs revision, especially those in the abstract and the introduction section.
2. The paragraph two, discussion section seems redundant. It can be removed and the contents may be written in relative paragraphs, respectively.

Minor points:

1. It is noted that the manuscript should be carefully edited to avoid grammar mistakes, e.g. the first sentence in paragraph 1, result section.
2. The left panel in figure 1A needs notes for symbols. E.g. the black dots represent mRNA level of wild type mSPARCL1 while grey dots represent that of SPARCL1 knockout type, which needs to be denoted clearly in the chart and/or figure legends.
